# Carotid sinus baroafferent signals contribute to cerebral blood flow regulation during acute hypotension in young males: A randomized crossover study

**DOI:** 10.14814/phy2.15937

**Published:** 2024-02-07

**Authors:** Kei Ishii, Tsubasa Izaki, Ryota Asahara, Hidehiko Komine

**Affiliations:** ^1^ Human Informatics and Interaction Research Institute, National Institute of Advanced Industrial Science and Technology Tsukuba Japan; ^2^ School of Economics & Management Kochi University of Technology Kochi Japan

**Keywords:** cerebral autoregulation, middle cerebral artery blood velocity, neck suction, neural regulation

## Abstract

Cerebral autoregulation is an important factor in prevention of cerebral ischemic events. We tested a traditional but unproven hypothesis that carotid sinus baroafferent signals contribute to dynamic cerebral autoregulation. Middle cerebral artery mean blood velocity (MCA V_mean_) responses to thigh‐cuff deflation‐induced acute hypotension were compared between conditions using neck suction soon after cuff deflation, without or with a cushion wrapped around the upper neck, in nine healthy males (aged 25 ± 5 years). Neck suction was applied close to the hypotension. The MCA V_mean_ response was expected to differ between conditions because the cushion was presumed to prevent the carotid sinus distension by neck suction. The cushion hindered bradycardia and depressor responses during sole neck suction. Thigh‐cuff deflation decreased mean arterial blood pressure (MAP) and MCA V_mean_ (*Ps* < 0.05) with an almost unchanged respiratory rate under both conditions. However, in the neck suction + cushion condition, subsequent MCA V_mean_ restoration was faster and greater (*Ps* ≤ 0.0131), despite similar changes in MAP in both conditions. Thus, carotid sinus baroafferent signals would accelerate dynamic cerebral autoregulation during rapid hypotension in healthy young males. Elucidating the mechanism underlying cerebral neural autoregulation could provide a new target for preventing cerebral ischemic events.

## 1 INTRODUCTION

The regulation of cerebral vasculature to maintain relatively stable blood flow in response to perfusion changes is called cerebral autoregulation (Claassen et al., 2021; Xiong et al., 2017). Cerebral autoregulation is strongly influenced by the rapidity of perfusion changes (i.e., steady‐state or rapid change) and is conceptually divided into two categories: static and dynamic autoregulation, respectively. Impaired cerebral autoregulation can result in cerebral blood flow reduction and washout failure of emboli during hypotension. Flow reduction and embolization seem to increase the risks of syncope, brain infarction, and subsequent cognitive impairment (Caplan et al., 2006; Howard et al., 2021; Murkin et al., 1997; Regenhardt et al., 2018; Zunker et al., 1998). Understanding the mechanism underlying cerebral autoregulation is thus important for preventing this cerebral ischemic cascade.

Overlapping mechanisms contribute to cerebral autoregulation (Brassard et al., 2023; Claassen et al., 2021); however, the role of neural mechanisms is unclear. The afferent signal from the carotid sinus baroreceptors is a possible contributor to cerebral “neural” autoregulation during rapid changes in arterial blood pressure (AP). This neural mechanism is reasonable particularly in humans, because AP in the brain is rapidly lowered due to the hydrostatic pressure gradients in the transition from a supine to a sitting or upright posture. Dynamic cerebral autoregulation, assessed by the autoregulatory index during rapid hypotension (White & Markus, 1997) and by transfer function parameters and correlation coefficient indices during spontaneous AP fluctuation (Hu et al., 1999; Reinhard et al., 2004), is impaired ipsilaterally in patients with carotid stenosis and is restored by carotid endarterectomy (Reinhard et al., 2004; White & Markus, 1997). These findings tempt us to believe a significant role of carotid sinus baroafferent signals in dynamic cerebral autoregulation. However, the changes in dynamic cerebral autoregulation might be attributed to the physical disturbance of arterial plaque. Arterial obstruction would lead to the development of collateral circulation and vasodilatation of cerebral arterioles, resulting in an impaired dilatory reserve capacity (Caplan et al., 2006; Reinhard et al., 2008). Hence, evidence is required to demonstrate dynamic cerebral autoregulation via the carotid sinus baroreceptors in healthy humans.

The possibility of involvement of a neural mechanism in dynamic cerebral autoregulation has also been reported by using systemic autonomic blockers. For example, trimethaphan, a systemic ganglion blocker, increased transfer function gain between AP and cerebral blood velocity fluctuations in the very‐low‐frequency range (0.02–0.07 Hz) and diminished the phase lead of cerebral blood velocity to AP (Zhang et al., 2002). Prazosin, a systemic α1‐adrenoreceptor blocker, accentuated the relative decrease in cerebral blood velocity during acute hypotension, resulting in a decrease in the rate of regulation (RoR) (Ogoh et al., 2008). These findings support the possibility that the autonomic nervous system plays a role in stabilizing cerebral blood flow. However, intravenous or oral administration of autonomic blockers affects systemic vasculature, as well as the central nervous system in some cases. This issue complicates interpretation of results previously obtained, i.e., whether the autonomic nervous system directly or indirectly (e.g., via AP changes) influences cerebral blood flow remains unclear. In addition, the possible effect of an intrinsic neural pathway (from the nucleus tractus solitarius [NTS] to higher brain regions) on cerebral blood flow regulation has been overlooked in the autonomic blocker experiments.

A nonpharmacological approach is needed to prove the role of a mechanism involving carotid sinus baroreceptors in dynamic cerebral autoregulation, irrespective of an intrinsic and extrinsic pathway. The carotid sinus baroreflex control of heart rate (HR) and sympathetic nerve activity during hypotension has been examined by using thigh‐cuff deflation with and without neck suction (Fadel et al., 2001). However, this conventional method is insufficient to isolate the possible role of carotid sinus baroafferent signals in dynamic cerebral autoregulation because perfusion pressure to the brain may be altered depending on the presence or absence of neck suction (which involves physical pressure). It is therefore essential to develop an appropriate control task that applies similar neck suction without distending the carotid sinus.

Accordingly, this study sought to establish a developed neck suction approach for examining dynamic cerebral neural autoregulation and to examine the presence or absence of dynamic cerebral neural autoregulation in healthy humans. By combining the developed neck suction and thigh‐cuff deflation methods, we non‐pharmacologically examined the traditional but unproven hypothesis that afferent signals from the carotid sinus baroreceptors contribute to the recovery of decreased cerebral blood flow during acute hypotension.

## 2 METHODS

The data from this study are available from the corresponding author upon reasonable request.

### 2.1 Participants

This crossover study was conducted referring to the CONSORT guidelines (Dwan et al., 2019) at the National Institute of Advanced Industrial Science and Technology. The sample size needed to test our hypothesis was calculated based on a previous study reporting the effect of systemic α1‐adrenergic blockade on dynamic cerebral autoregulation (Ogoh et al., 2008). The estimated sample size was nine when setting the type I error probability and statistical power at 0.05 and 0.95, respectively. However, due to dropouts (see *Data treatment and statistical analyses* for details), 21 male participants were recruited from a portal site for college students. Inclusion criteria included 18 years ≤ age ≤ 35 years; no history of cardiovascular, cerebrovascular, or neuromuscular disease; and non‐smokers. Male participants were purposely recruited in the main experiment because of our previous experience that AP was insufficiently decreased by thigh‐cuff deflation in female participants. In another experiment (see *Protocols* for details), 10 other participants (five females; age, 40 ± 11 years; height, 163 ± 13 cm; weight, 58 ± 13 kg; means ± SD) were recruited.

This study was approved by the Institutional Review Board of the National Institute of Advanced Industrial Science and Technology (HF2016‐0322). The participants provided written informed consent for their participation and this publication, and were asked to abstain from drinking alcohol and caffeine for 24 h before the experiments.

### 2.2 Measurements

**FIGURE 1:**
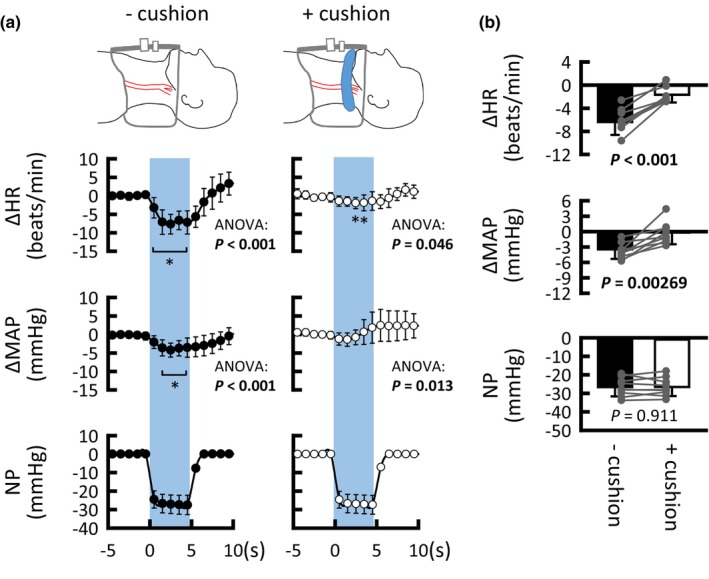
Effects of sole neck suction with or without cushion on heart rate (HR) and mean arterial blood pressure (MAP). (a) Time courses of the averaged HR and MAP responses to neck suction with (○) and without cushion (●). (b) Comparison of HR, MAP, and induced neck pressure (NP) between neck suction with (□) and without cushion (■) conditions. Gray circles indicate individual data (*n* = 9). *p* < 0.05 are in bold. Values are means ± SD. *Significant difference (*p* < 0.05) compared with the control values.

An electrocardiogram was monitored with a telemetry system (BSM‐2401, Nihon Kohden, Tokyo, Japan) to measure HR. AP was continuously measured using finger photoplethysmography (Finometer Pro; Finapres Medical Systems, Amsterdam, The Netherlands) at a frequency of 200 Hz. Exhaled breath temperature was measured using a nasal temperature probe (MLT415, ADInstruments, Sydney, Australia) for calculating the respiratory rate (RR). In one participant, RR and end‐tidal carbon dioxide (CO_2_) were measured using a capnometer (OLG‐3800, Nihon Kohden). Middle cerebral artery (MCA) blood velocity was measured at a frequency of 100 Hz with transcranial Doppler (TCD) ultrasonography (Doppler‐BoxTM X, DWL, Sipplingen, Germany). A 2‐MHz Doppler probe was placed over the left temporal window and fixed with an adjustable headband, and the data of MCA blood velocity was recorded at a depth of 47–54 mm. The tissue oxygenation index (TOI) of the forehead was measured at a frequency of 5 Hz using near‐infrared spectrometry (NIRS; NIRO‐200NX, Hamamatsu Photonics, Hamamatsu, Japan) to estimate blood flow responses in the prefrontal cortex as reported previously (Al‐Rawi et al., 2001; Ishii et al., 2018). The photoemission and detection probes were placed on the surface of the left forehead. The interprobe distance was 4 cm. To monitor the possible influence of skin blood flow (SBF) on the TOI signal, SBF was measured on the left infraorbital region by using a laser Doppler flowmeter (FLO‐C1, OMEGAWAVE, Tokyo, Japan) with a time constant of 0.1 s. A malleable collar with or without a flexible cushion was wrapped around the neck to apply subatmospheric pressure within the chamber (Figure 1). The cushion was dumbbell‐shaped (2 ellipsoids: short axis × long axis = 7 cm × 14 cm) and consisted of a plastic wrap‐covered latex glove stuffed with polyethylene cushioning beads. The materials were selected (1) to allow the cushion to fit softly around the individual's neck and (2) to restrict overextension of the cushion by neck suction. The cushion was closely wrapped around the upper neck and was stabilized by a 1‐kg weight. Pressure within the chamber was measured with a pressure transducer (AP‐C30, KEYENCE, Osaka, Japan).

### 2.3 Experimental strategy using the newly developed neck suction approach

This study consisted of two experimental steps. First, we attempted to verify the developed approach of applying neck suction with and without a flexible cushion wrapped around the upper neck (i.e., at the location of the carotid sinus). Conventional neck suction without a cushion (−cushion) stimulates the baroreflex by distending the carotid sinus (Kober & Arndt, 1970). On the other hand, we assumed that neck suction with the cushion (+cushion) caused distension of the common carotid arteries while preventing distension of the carotid sinuses. Then, cerebral blood flow responses to acute hypotension caused by thigh‐cuff deflation were compared between neck suction ± cushion conditions, to extract the effect of carotid sinus baroafferent signals on cerebral blood flow regulation during rapid hypotension. That is, when neck suction − cushion was applied close to the changes in mean AP (MAP) caused by thigh‐cuff release, the neck suction would maintain the diameter of the carotid sinus against the hypotension (i.e., maintain the carotid sinus baroafferent nerve activity at the pre‐hypotension level) and prevent or weaken the carotid sinus baroreflex to hypotension (Fadel et al., 2001; Sanders et al., 1989). In contrast, when neck suction + cushion was similarly applied, we assumed that the carotid sinus baroreceptors could detect the hypotension in the state of similar negative pressure to the exposed neck, and thereby resulting in inhibition of carotid sinus baroafferent nerve activity and subsequent activation of the carotid sinus baroreflex. Therefore, the physical influence of neck suction on perfusion pressure to the brain was assumed to be similar between the neck suction ± cushion conditions. Comparison of the cerebral blood flow responses between these conditions would therefore enable to isolate the possible role of carotid sinus baroafferent signals in dynamic cerebral autoregulation. We monitored constant end‐tidal CO_2_ during sole neck suction and during the 20 s from the onset of thigh‐cuff deflation in one participant. In addition, we evaluated whether the negative pressure applied on the proximal tissues (e.g., neck suction) physically altered perfusion pressure in the distal arteries.

### 2.4 Protocols

The participants rested for approximately 10 min in a supine position on a bed after they were instrumented. The participants were asked to relax and avoid breath‐holding throughout the experiments. We first determined the optimal level of neck suction that caused nadir HR reduction by ca. 10–15 beats/min by applying negative pressure to various degrees in each participant. Using the optimal negative pressure, sole neck suction for 5 s was applied 5–6 times with or without a cushion, to certainly examine whether the carotid sinus baroreflex (baroreflex bradycardia and hypotension) was stimulated, as shown in Figure 1. The sole neck suction trial was performed before each thigh‐cuff deflation trial.

Thereafter, to examine the effect of carotid sinus baroafferent signals on cerebral blood flow during acute hypotension, bilateral thigh cuffs were inflated to 200 mmHg, maintained inflated for 3 min, and then deflated rapidly by a rapid cuff inflation–deflation system (E20, AG101, and CC17TM, D. E. Hokanson, Bellevue, WA, USA). This step was repeated three times with the following modifications. In the first trial, deflation without any intervention was performed to familiarize the participant with the trial and confirm the peak decrease in MAP. In the second trial, neck suction − cushion was applied manually within the chamber close to the MAP changes evoked by thigh‐cuff deflation. The neck suction was applied for 1 min from the onset of cuff deflation. In the third trial, neck suction + cushion was applied during deflation in a similar manner. Each trial was followed by a 15‐min rest period. The order of the second and third trials was randomized by a computer in a counterbalanced crossover manner. A 3‐min inflation of thigh‐cuff was chosen because we sought to decrease the unpleasant period due to thigh‐cuff inflation as much as possible and because this duration was sufficient to induce hypotension and is also used generally (e.g., Ogoh, Sato et al., 2010). The participants were asked to rate their discomfort from 0 (not unpleasant at all) to 10 (the most unpleasant) after sole neck suction trials and thigh‐cuff deflation trials.

On a separate day, another experiment was performed to evaluate the possibility of negative pressure on proximal tissues physically altering the perfusion pressure to distal arteries. We examined whether negative pressure on the arm (instead of the neck) physically changed AP measured at the left middle finger in the 10 other participants. The arm was placed in a customized polystyrene box, positioning the elbow around the center. Arm suction ± cushion (at the distal end of the arm in the box) for 10 s was applied 2–3 times on the side ipsilateral to the finger used for AP measurement. The same procedure, without a cushion, was also applied on the contralateral arm.

### 2.5 Data treatment and statistical analyses

All data were stored in a computer at a sampling frequency of 1 kHz for off‐line analysis, and information for the participants was masked by a technical staff‐member. A data analyst was kept blinded to the trial order until statistical analysis.

The onset of cuff deflation was defined as “time = 0.” The beat‐to‐beat values of MAP and MCA mean blood velocity (V_mean_) were calculated from each waveform. The cerebrovascular conductance index (CVCi) was calculated by dividing MCA V_mean_ by MAP to estimate changes in cerebrovascular conductance. Skin vascular conductance (SVC) was similarly calculated. Control values were defined as averages during the 10 s before sole neck suction or thigh‐cuff deflation. Percentage changes in MAP, MCA V_mean_, CVCi, SBF, and SVC relative to their control values were calculated. Changes in all variables were sequentially averaged every 1 s. Of 21 participants, two discontinued the experiments due to discomfort caused by thigh‐cuff inflation. Two slept during thigh‐cuff inflation. In eight participants, sufficient hypotension (ΔMAP <−10 mmHg) and/or baroreflex tachycardia did not occur by thigh‐cuff deflation, as has been reported previously (White & Markus, 1997). Therefore, we analyzed data of the remaining nine male participants (age, 25 ± 5 years; height, 172 ± 6 cm; body weight, 65 ± 4 kg). The order of trials was counterbalanced in these participants.

The control values were compared between conditions by using a paired *t*‐test or Wilcoxon rank‐sum test. In sole neck suction trials, HR and MAP responses were analyzed by a one‐way repeated‐measures ANOVA or Friedman ANOVA with a Dunnett's post hoc test. Then, the HR and MAP responses and applied neck pressure were averaged for 5 s and compared between conditions using a paired *t*‐test. In thigh‐cuff deflation trials, we focused on 1–8 s after the onset of thigh‐cuff deflation because MAP remained at a low nadir for 6–8 s (Ogoh et al., 2008), thereby minimizing the effect of increasing perfusion pressure on the recovery of MCA V_mean_. Changes in each variable from control values during 1–8 s from thigh‐cuff deflation were analyzed by a one‐way repeated‐measures ANOVA or Friedman ANOVA with a Dunnett's post hoc test. The initial period was divided into two (period I, from 1 to 4 s; period II, from 4 to 8 s). Period I almost corresponds to the period for evaluating the RoR, which is believed to be related to dynamic cerebral autoregulation without baroreflex regulation (Aaslid et al., 1989). The RoR in the preset time range was calculated based on the following equation:

$$\mathrm{RoR}=\left(\text{Δrelative CVCi}/\mathrm{\Delta T}\right)/\text{Δrelative}\;\mathrm{MAP}.$$

where (Δrelative CVCi/ΔT) is the slope of the linear regression between relative CVCi and time (T, 1.0–3.5 s from thigh‐cuff deflation), and Δrelative MAP is the averaged relative changes in MAP during the same time period (T) (Aaslid et al., 1989; Ogoh et al., 2008). In addition, the onset of cerebrovascular counterregulation (i.e., when CVCi starts to recover) was evaluated and was then used to calculate another RoR value during a 2.5‐s interval starting after the individually determined onset of cerebrovascular counterregulation as previously reported (Labrecque et al., 2017; Lind‐Holst et al., 2011). Averaged changes in all variables during the two periods and RoR values were compared between neck suction ± cushion conditions using a paired *t*‐test or Wilcoxon rank‐sum test with Bonferroni correction. We used the averaged cerebral blood flow responses to perfusion pressure changes in the periods as a concept‐based index of dynamic cerebral autoregulation and the primary outcome of this study, because the main outcome of dynamic cerebral autoregulation is a cerebral blood flow response to the perfusion pressure changes at a time point or within a period as described in the *Introduction*. The primary outcome was described with the 95% CI difference between the mean and estimated effect size (*r*). Since cardiac output likely affects cerebral blood flow (Ogoh & Tarumi, 2019; Ogoh, Tzeng et al., 2010), an increase in HR may also contribute to MCA V_mean_ responses to thigh‐cuff deflation. Using pooled data of neck suction ± cushion conditions, the relationships of tachycardiac responses with the RoR values or MCA V_mean_ responses were examined in each period, using Pearson's correlation analysis with Bonferroni correction. The reported levels of discomfort were compared between conditions using a Wilcoxon rank‐sum test.

We estimated the physical effects of pressure applied to proximal tissues on perfusion pressure to distal arteries in two ways. First, the averaged cerebral blood flow, HR, and MAP responses during periods I and II after thigh‐cuff deflation were compared between the neck suction + cushion condition and the no‐neck suction condition (i.e., 1st trial) using a paired *t*‐test. The MCA V_mean_ response was also analyzed using a one‐way ANCOVA with the control MAP values and/or the MAP responses as covariates. If neck suction + cushion altered cerebral perfusion pressure, a significant difference in cerebral blood flow responses would be observed. Second, the physical effects of arm suction on the finger AP were analyzed by a one‐way repeated‐measures ANOVA or Friedman ANOVA with a Dunnett's post hoc test. Moreover, the averaged HR and AP responses during 1–4 s and 4–8 s of arm suction (to align with periods I and II) were compared between ±cushion conditions and between ipsilateral and contralateral arm suctions using a one‐way repeated‐measures ANOVA or Friedman ANOVA. All statistical analyses were conducted using SigmaPlot version 14.0 (Systat Software, San Jose, CA, USA). The statistical significance level was defined as *p* < 0.05 or was corrected with the Bonferroni method (*p* < 0.025) when necessary. All data values are expressed as means ± SD.

## 3 RESULTS

### 3.1 Sole neck suction trials

The control values of HR and MAP were not significantly different with or without cushion conditions (59 ± 10 versus 60 ± 8 beats/min, *p* = 0.615; 89 ± 8 versus 87 ± 9 mmHg, *p* = 0.07, respectively). Figure 1 shows the averaged HR and MAP responses to neck suction with and without a cushion. Neck suction without a cushion decreased HR and MAP, whereas neck suction with a cushion caused only a slight decrease in HR. The cushion clearly decreased (*p* ≤ 0.00269) the effect of neck suction on HR and MAP (Figure 1b), even though similar neck pressure was applied (*p* = 0.911). Discomfort scores were similar without (5.7 ± 1.3) and with the cushion (5.4 ± 1.2; *p* = 0.578).

### 3.2 Thigh‐cuff deflation trials

**FIGURE 2:**
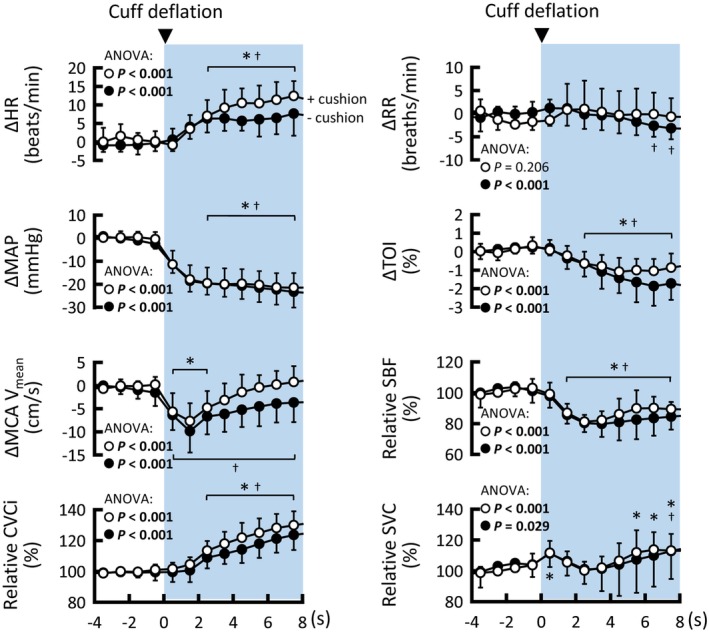
Time courses of the averaged cardio‐ and cerebrovascular responses to thigh‐cuff deflation with neck suction + cushion (○) and −cushion (●) (*n* = 9). SBF, CVCi, and SVC data are shown in normalized units relative to the control values. In two participants in neck suction + cushion, TOI data were lack due to probe‐adhesive failure. *p* < 0.05 are in bold for ANOVA. Values are means ± SD. HR, heart rate; MAP, mean arterial blood pressure; MCA V_mean_, mean blood velocity of the middle cerebral artery; CVCi, cerebrovascular conductance index; RR, respiratory rate; TOI, tissue oxygenation index; SBF, skin blood flow; SVC, skin vascular conductance. *Significant difference (*p* < 0.05) compared with control values in neck suction ± cushion condition. ^†^Significant difference (*p* < 0.05) compared with the control values in neck suction − cushion conditions.

HR and MAP before thigh‐cuff inflation were similar between neck suction + and −cushion conditions (60 ± 10 versus 59 ± 8 beats/min, *p* = 0.667; 90 ± 9 versus 87 ± 10 mmHg, *p* = 0.0532, respectively). Control values were not significantly different between neck suction ± cushion conditions (Table 1). Cardio‐ and cerebrovascular responses to thigh‐cuff deflation with neck suction ± cushion are summarized in Figure 2. The thigh‐cuff was deflated in the similar respiratory phase in both conditions (from expiration‐onset, 1.1 ± 0.7 s in neck suction − cushion [*n* = 4] and 1.1 ± 0.4 s in neck suction + cushion [*n* = 5]; from inspiration‐onset, 0.8 ± 0.5 s in neck suction − cushion [*n* = 4] and 1.0 ± 0.2 s in neck suction + cushion [*n* = 3]). The timing of neck suction was also similar between conditions (*p* = 0.652).

**TABLE 1:** Control values of hemodynamic and respiratory variables.

	Neck suction − cushion	Neck suction + cushion	No‐neck suction	*p*‐Value^a^	*p*‐Value^b^
HR (beats/min)	62 ± 9	63 ± 7	61 ± 7	0.4069	0.103
MAP (mmHg)	97 ± 10	100 ± 7	94 ± 7	0.0902	**0.004457**
RR (breaths/min)	18 ± 7	17 ± 6	16 ± 6	0.3657	0.5396
MCA V_mean_ (cm/s)	54 ± 17	53 ± 14	51 ± 16	0.543	0.302
CVCi (cm/s/mmHg)	0.60 ± 0.20	0.53 ± 0.15	0.54 ± 0.17	0.05373	0.7555
TOI (%)	67 ± 7	68 ± 7	66 ± 9	0.3883	0.07959
SBF (mL/min/100 g)	9.3 ± 7.2	7.0 ± 2.9	9.7 ± 7.2	0.8203	0.4961
SVC (mL/min/100 g/mmHg)	0.10 ± 0.07	0.07 ± 0.04	0.11 ± 0.08	0.9102	0.4961

*Note*: Values are means ± SD. *p* < 0.05 are in bold.

Abbreviations: CVCi, cerebrovascular conductance index; HR, heart rate; MAP, mean arterial pressure; MCA V_mean_, mean blood velocity of the middle cerebral artery; RR, respiratory rate; SBF, skin blood flow; SVC, skin vascular conductance; TOI, tissue oxygenation index.

^a^ *p*‐value expresses the results of paired *t*‐test between neck suction ± cushion conditions.

^b^ *p*‐value expresses the results of paired *t*‐test between neck suction + cushion and no‐neck suction conditions.

Thigh‐cuff deflation in both neck suction ± cushion conditions caused a rapid MAP decrease and a reflex HR increase, with RR remaining virtually unchanged. A transient decrease in MCA V_mean_ was caused by thigh‐cuff deflation; the decreased MCA V_mean_ was recovered to the control level at 4 s from thigh‐cuff deflation in the neck suction + cushion condition, whereas MCA V_mean_ did not recover during 8 s in the neck suction − cushion condition. The relative CVCi increased at 3 s in both conditions. The decreases in MAP and MCA V_mean_ reached the nadir at a similar timing between neck suction ± cushion conditions (−cushion versus + cushion; 6.7 ± 3.6 versus 6.5 ± 3.2 s in MAP, *p* = 0.878; 0.6 ± 0.1 versus 0.8 ± 0.3 s in MCA V_mean_, *p* = 0.172). The nadir decrease in MAP was greater in the neck suction − cushion condition (71 ± 10 versus 76 ± 7 mmHg, *p* = 0.0323), whereas that in MCA V_mean_ was similar between the two conditions (−cushion versus + cushion; 43 ± 14 versus 45 ± 12 cm/s, *p* = 0.427). In both conditions, the prefrontal TOI decreased at 3 s from thigh‐cuff deflation, facial SBF decreased at 2 s, and SVC remained close to the control level but increased slightly. Discomfort scores were similar between the conditions (neck suction − cushion, 7.1 ± 1.2; neck suction + cushion, 7.3 ± 1.3; *p* = 0.626).

**FIGURE 3:**
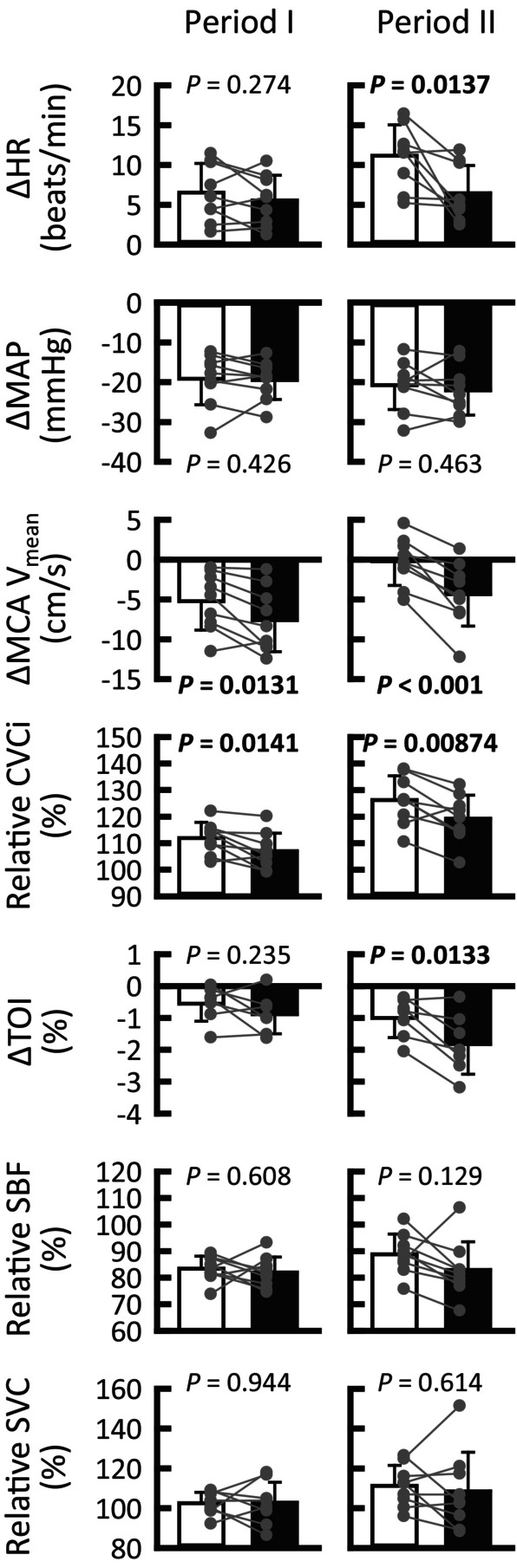
Averaged cardio‐ and cerebrovascular responses in periods I (1–4 s) and II (4–8 s) following the onset of thigh‐cuff deflation. The neck suction − cushion (■) sought to prevent or weaken the carotid sinus baroreceptor responses to hypotension by distending the carotid sinus area, while neck suction + cushion (□) allowed the baroreceptors to respond to hypotension in the state of similar negative pressure to the exposed neck. Gray circles indicate individual data (*n* = 7 for TOI and *n* = 9 for others). Significance level was defined as *p* < 0.025 after Bonferroni correction. *p* < 0.025 are in bold. Values are means ± SD. HR, heart rate; MAP, mean arterial blood pressure; MCA V_mean_, mean blood velocity of the middle cerebral artery; CVCi, cerebrovascular conductance index; TOI, tissue oxygenation index; SBF, skin blood flow; SVC, skin vascular conductance.

**FIGURE 4:**
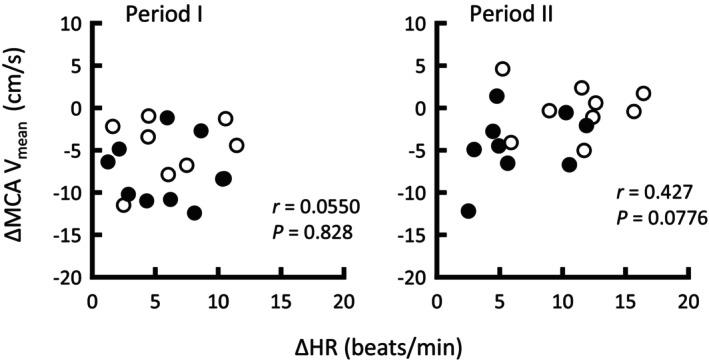
Relationship between changes in HR and MCA V_mean_ in periods I and II following the onset of thigh‐cuff deflation. Data in neck suction − cushion (●) and +cushion (○) conditions were pooled (*n* = 9). Significance level was defined as *p* < 0.025 after Bonferroni correction. HR, heart rate; MCA V_mean_, mean blood velocity of the middle cerebral artery.

Figure 3 summarizes the effects of neck suction ± cushion on cardio‐ and cerebrovascular responses to thigh‐cuff deflation. We first confirmed that neither neck pressure nor estimated common carotid artery pressure (calculated by subtracting applied chamber pressure from MAP) differed (*Ps* ≥ 0.354) between the conditions. During period I (1–4 s), the decrease in MCA V_mean_ was smaller (*p* = 0.0131, *r* = 0.75, 95% CI difference: 0.641–4.044) and the increase in relative CVCi was greater (*p* = 0.0141) in the neck suction + cushion than in the neck suction − cushion condition. Changes in all other variables were not significantly different between conditions. Time delay of cerebrovascular counterregulation in the neck suction + cushion condition (1.7 ± 0.4 s) was comparable to that in the neck suction − cushion condition (1.8 ± 0.4 s; *p* = 0.312). RoR in the individually determined time range revealed a significant difference between conditions (+cushion, 0.267 ± 0.130/s; −cushion, 0.0982 ± 0.134/s; *p* = 0.0162), while RoR in the preset time range (1.0–3.5 s) tended to be greater (*p* = 0.085) in the condition with (0.375 ± 0.156/s) than in the condition without a cushion (0.281 ± 0.124/s). During period II (4–8 s), decreases in MCA V_mean_ and prefrontal TOI were smaller (*p* < 0.001, *r* = 0.88, 95% CI difference: 2.323–5.932 and *p* = 0.0133, respectively) and the increase in CVCi was greater (*p* = 0.00874) for neck suction + cushion. The decrease in MAP was similar (*p* = 0.463) between conditions, while the increase in HR became greater (*p* = 0.0137) in the neck suction + cushion condition. There were no significant differences in facial SBF and SVC responses between the conditions. Figure 4 shows that the HR response to thigh‐cuff deflation was unrelated to the MCA V_mean_ response in both periods I (*p* = 0.828) and II (*p* = 0.0776). Similarly, no relationship between the HR response and each RoR was observed (*p* = 0.648 in the individually determined time range; *p* = 0.255 in the preset time range).

**FIGURE 5:**
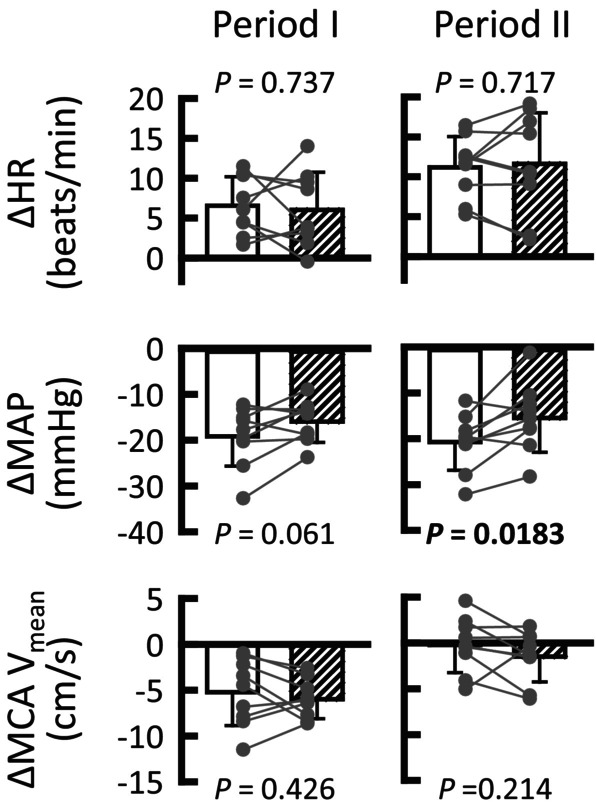
Effect of neck suction + cushion on MCA V_mean_ responses in periods I (1–4 s) and II (4–8 s) following the onset of thigh‐cuff deflation. Gray circles indicate individual data (*n* = 9). The significance level was defined as *p* < 0.025 after Bonferroni correction. *p* < 0.025 are in bold. Values are means ± SD. Open bars, neck suction + cushion condition; diagonal line bars, no‐neck suction condition. HR, heart rate; MAP, mean arterial blood pressure; MCA V_mean_, mean blood velocity of the middle cerebral artery.

The cardio‐ and cerebrovascular responses to thigh‐cuff deflation were also compared between neck suction + cushion and no‐neck suction conditions to estimate the physical effects of pressure applied to the neck on perfusion pressure in the brain (Figure 5). The control HR and MCA V_mean_ were similar between conditions, while the control MAP was slightly increased in the neck suction + cushion condition (Table 1). The MCA V_mean_, HR, and MAP responses did not differ between conditions in period I (*p* = 0.426, 0.737, and 0.061, respectively). Similar results were observed in period II, although the MAP response differed between conditions (*p* = 0.0183). One‐way ANCOVA, in which the control MAP and the MAP response were covariates, also revealed no significant difference between the adjusted means of the MCA V_mean_ response in both periods I (*p* = 0.514) and II (*p* = 0.247).

### 3.3 Arm suction trials

**FIGURE 6:**
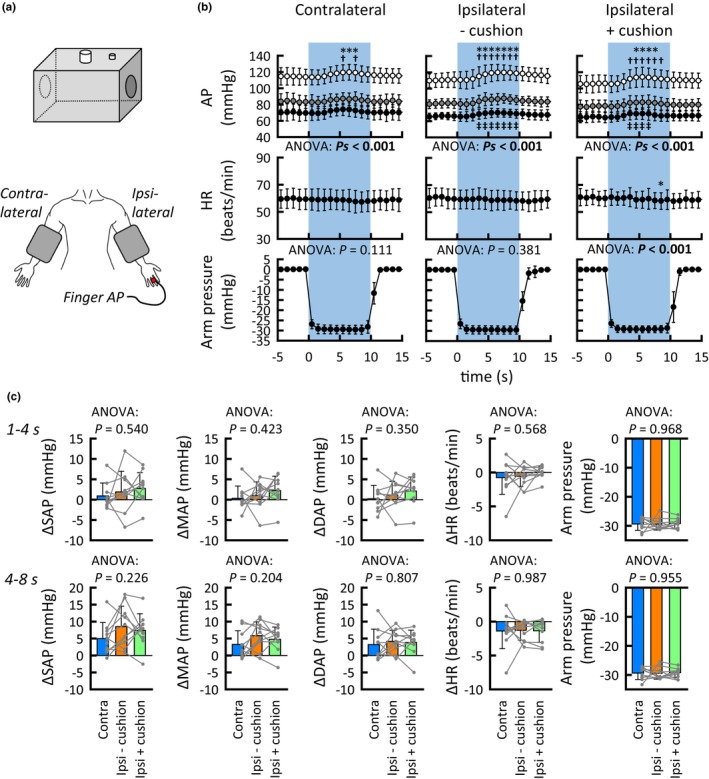
Effects of arm suction on the finger AP. (a) Experimental setting of arm suction. Negative pressure was applied on the arm of ipsilateral or contralateral side to the AP‐measured finger. Ipsilateral arm suction was conducted with and without cushion. (b) Time courses of the averaged HR and AP (SAP [○], MAP [●], and DAP [●]) responses to three types of arm suction (*n* = 10). *Significant changes (*p* < 0.05) from the rest of SAP or HR; ^†^Significant changes (*p* < 0.05) from the rest of MAP; ^‡^Significant changes (*p* < 0.05) from the rest of DAP. *p* < 0.05 are in bold for ANOVA. (c) The averaged HR and AP responses during 1–4 s and 4–8 s of arm suction (*n* = 10). Gray circles indicate individual data. Values are means ± SD. AP, arterial blood pressure; SAP, systolic AP; MAP, mean AP; DAP, diastolic AP; Contra, contralateral arm suction; Ipsi − cushion, ipsilateral arm suction without cushion; Ipsi + cushion, ipsilateral arm suction with cushion.

Figure 6 summarizes the physical effects of negative pressure applied to proximal tissues (the arm) on perfusion pressure to the distal arteries (AP at the finger). Arm suction ± cushion had no effect on the finger AP until 4–5 s from the suction onset (Figure 6b). Thereafter, slight pressor responses occurred in all the arm suction conditions. The cardiovascular responses during 1–4 s and 4–8 s of arm suction were similar between conditions (ANOVA: *Ps* ≥ 0.204).

## 4 DISCUSSION

This study investigated the direct impact of carotid sinus baroafferent signals on decreased cerebral blood flow during acute hypotension in healthy young males. The newly developed approach (neck suction with and without a cushion) revealed that the cardiovascular responses caused by neck suction were hindered by the cushion wrapped around the upper neck. Using this approach and thigh‐cuff deflation, we found that the transient decrease in MCA V_mean_ caused by acute hypotension was recovered more rapidly in the neck suction + cushion than in the neck suction − cushion condition. Moreover, we attempted to disclose the physical effect of negative pressure applied to proximal tissue on perfusion pressure to the distal tissues in two ways. The results obtained from the comparison of the MCA V_mean_ responses between neck suction + cushion and no‐neck suction conditions and from the arm suction trials raise a possibility that the proximal negative pressure may have a minor effect on the distal perfusion pressure. Taken together, the present findings demonstrated a functional role of carotid sinus baroafferent signals in dynamic cerebral autoregulation during acute hypotension in healthy young males.

Whether cerebral “neural” autoregulation exists has been a long‐standing unresolved issue. In animals, conflicting results regarding the issue were reported because of differences in animal species and conditions, methodology and timing of cerebral blood flow measurement, and experimental manipulation (Heistad & Marcus, 1976; Heistad & Marcus, 1978; Nakai & Ogino, 1984; Sagawa & Guyton, 1961). In humans, the possible role of the carotid sinus baroafferent signals in dynamic cerebral autoregulation was reported along two lines. First, the dynamic autoregulation is impaired by carotid stenosis and restored by carotid endarterectomy (Hu et al., 1999; Reinhard et al., 2004; White & Markus, 1997). However, the possibility cannot be excluded that carotid stenosis may lead to a reduction in the vasodilatory reserve capacity, which may be solved after the procedure. Second, the dynamic autoregulation is affected by systemic sympathetic, parasympathetic, and ganglionic blockers (Hamner et al., 2010; Hamner et al., 2012; Ogoh et al., 2008; Zhang et al., 2002). However, systemic autonomic blockade influencing systemic vasculature complicates the results of relative cardio‐ and cerebrovascular responses against different baseline values, as does separating the direct and indirect effects of the autonomic nervous system on cerebral blood flow (Ogoh & Tarumi, 2019). Therefore, using a nonpharmacological approach in healthy participants is important to verify the direct contribution of the carotid sinus baroafferent signals to dynamic cerebral autoregulation.

Conventional neck suction/pressure methods can manipulate the carotid sinus baroreceptor loading (Eckberg, 1976; Kober & Arndt, 1970). We developed a new method, where we used neck suction with and without a flexible cushion wrapped around the upper neck, assuming that the cushion would inhibit baroreflex responses to distension of the carotid sinus caused by neck suction (Figure 1). However, a comparison of the MCA V_mean_ response between neck suction with and without a cushion would be insufficient to confirm “direct” neural regulation of cerebral blood flow, because an indirect effect of the evoked AP and HR responses on cerebral circulation cannot be disregarded.

To examine the direct contribution of carotid sinus baroafferent signals to dynamic cerebral autoregulation, the MCA V_mean_ response to thigh‐cuff deflation was compared between neck suction ± cushion conditions. During period I (1–4 s), we found that neck suction − cushion, i.e., inhibiting the carotid baroreceptor deactivation, delayed the recovery of MCA V_mean_ as compared to neck suction + cushion, despite similar HR, MAP, and RR responses between conditions (Figures 2 and 3). The results strongly suggest that carotid sinus baroafferent signals contributed directly to cerebral autoregulation in the face of rapid hypotension. The rapid recovery of cerebral blood flow would result from rapid vasodilatation of the MCAs and/or distal arteries because of the greater increase in relative CVCi in the neck suction + cushion condition. Similar results were evident during period II (4–8 s). The averaged MCA V_mean_ was restored from the nadir by 63% in the neck suction + cushion and 44% in the neck suction − cushion condition at 4 s and 109% and 67% at 8 s, indicating that approximately 20%–40% of dynamic cerebral autoregulation is derived from the carotid sinus baroafferent signals. We noted that cardiac acceleration may play a minor role, if any, in the recovery of MCA V_mean_, because the HR response to thigh‐cuff deflation did not correlate with the MCA V_mean_ response in both periods I and II (Figure 4), or the RoR values. Moreover, a concern that differences in subjective feelings to neck suction with and without a cushion might result in differential MCA V_mean_ responses was found to be invalid, because similar discomfort scores were reported with or without the cushion. Taking these results into account, it is likely that the carotid sinus baroafferent signal is functionally important for dynamic cerebral autoregulation from period I of rapid hypotension. However, these findings may not be simply generalized to cases with rapid hypertension (Labrecque et al., 2022; Panerai et al., 2023).

Afferent signals from the carotid sinus baroreceptors would regulate cerebral blood vessels via the intrinsic and/or extrinsic pathways from the NTS. In the intrinsic pathway, the NTS neurons project to the broad brain areas (Kawai, 2018), and the directly/indirectly projected neurons may regulate cerebral blood vessels via intracranial fibers. For instance, the NTS neurons project to the locus coeruleus which projects to the nucleus basalis of Meynert (NBM). The locus coeruleus neurons affect cerebral arterioles and capillaries via perivascular astroglial leaflets (Bekar et al., 2012; Cohen et al., 1997), while the NBM directly innervates parenchymal arteries and arterioles (Hotta et al., 2013). Furthermore, activation or inhibition of the NTS neurons would cause activation or inhibition of neurons projected from the NTS. The sequence of neural activity would cause neurovascular coupling responses around the projected neurons (Hosford & Gourine, 2019). The extrinsic pathway runs via the peripheral nervous system (sympathetic, parasympathetic, and sensory nerves) innervating the cerebral blood vessels (Briggs et al., 1985; Edvinsson et al., 1976; Suzuki et al., 1989). Any of these pathways can directly impact dynamic cerebral autoregulation.

The effect of carotid sinus baroafferent signals on cerebral blood vessels may differ regionally: the MCA V_mean_ response differed between conditions in periods I and II, whereas the prefrontal TOI response differed only in period II (Figures 2 and 3). The prefrontal TOI response did not appear to be influenced by SBF responses. These results may mean that the baroafferent signals started to assist recovery of prefrontal blood flow from period II. However, this is speculative and is based on different methodologies for evaluating cerebral blood flow responses (TCD versus NIRS). A comparison of blood velocity responses of the MCA and anterior cerebral artery is needed. The same caution is true regarding the posterior cerebral artery.

A major caveat to consider when interpreting the present findings is the physical effect of neck suction on the cerebral perfusion pressure. We inferred that any putative effect would be a minor, for the following reasons. First, negligible baroreflex cardiovascular responses to sole neck suction + cushion (Figure 1) indicate that sole neck suction, at least in the +cushion condition, would not change perfusion pressure to the carotid sinuses and more distal arteries. Second, it might be possible that thigh‐cuff deflation with neck suction ± cushion might result in different cerebral perfusion pressure between conditions because of the different MCA V_mean_ responses (in the branch from the internal carotid artery) during periods I and II between conditions (Figures 2 and 3); however, the SBF responses (in branches from the external carotid artery) during the periods were similar between conditions. It is unlikely that a physical effect of neck suction on distal perfusion pressure differed between the internal and external carotid arteries. Third, one‐way ANCOVA revealed that neck suction + cushion had no influence on the MCA V_mean_ responses to thigh‐cuff deflation in comparison to that without any intervention (Figure 5). Finally, arm suction ± cushion had no influence on the finger AP until 4–5 s from the onset of arm suction (Figure 6). Thereafter, the startle‐like pressor response similarly occurred during any of the arm suction experiments, regardless of whether a cushion was used and which side was suctioned (Figure 6c). These findings imply that perfusion pressure to the brain would not be physically influenced by neck suction. This is a rationale‐based notion, but the actual perfusion pressure remains unknown. However, comparing the MCA V_mean_ responses between neck suction pressure ± cushion conditions is likely at least to cancel out both physical and startle‐like effects of neck suction on the cerebral perfusion pressure.

Another caveat is that the cardiovascular responses to sole neck suction + cushion (Figure 1) are still debatable, because of the slight change in HR by −2 beats/min. One explanation for this slight bradycardia is that the cushion could not perfectly block the effect of neck suction (i.e., carotid sinus baroreflex). This notion might be supported by the finding that the cuff deflation‐induced hypotension was not similar, but was rather greater in the neck suction + cushion than in the no‐neck suction condition (Figure 5); however, the different hypotension levels (e.g., in period II, −15 ± 7 mmHg in no‐neck suction vs. −21 ± 6 mmHg in neck suction + cushion) seems to be more explainable by the difference in control MAP by ca. 6 mmHg (Table 1), and the possible effect of neck suction + cushion was not sufficient to extract the effect of carotid sinus baroafferent signals on the MCA V_mean_ response (Figure 5). In such a case, comparing the cardio‐ and cerebrovascular responses between conditions might underestimate the role of carotid sinus baroafferent signals in the regulation of the cardio‐ and cerebrovascular systems. Another possibility is that neck suction induces startle‐like cardiovascular responses. Both arm suction and neck suction + cushion appeared to cause similar startle‐like cardiovascular responses (Figures 1 and 6b). Even if this was the case, we succeeded in canceling the startle‐like effect on cardio‐ and cerebrovascular responses by comparing between neck suction ± cushion conditions.

This study had several limitations. First, as reported previously (Ogoh et al., 2008; Ogoh, Tzeng et al., 2010; Zhang et al., 2002), the MCA V_mean_ is regarded as a surrogate for cerebral blood flow because direct observation of the MCA diameter shows unresponsiveness (ca.0.14%/mmHg) to moderate changes in AP (0–40 mmHg) (Giller et al., 1993). Even if the diameter was changed by hypotension, differential MCA V_mean_ responses between the conditions (Figures 2 and 3) suggest that carotid sinus deactivation by hypotension would regulate the MCA diameter rapidly. Second, intracranial pressure and central venous pressure (determinants of cerebral perfusion pressure) were not measured in this study, although (1) central venous pressure was unaltered after thigh‐cuff deflation with or without neck suction (Fadel et al., 2001) and (2) combining the previous finding and the present result of the similar MCA V_mean_ responses adjusted by MAP responses (Figure 5) leads us to speculate that intracranial pressure responses in thigh‐cuff deflation trials might be also similar across all conditions. Third, we did not measure the partial pressure of arterial or end‐tidal CO_2_, which can influence the diameter of cerebral blood vessels (Coverdale et al., 2015; Hotta et al., 2013). However, the participants were asked to breathe as usual and to avoid breath‐holding. Thigh‐cuff deflation was evoked in the similar respiratory phase between neck suction ± cushion conditions and did not induce a RR change during period I (Figure 2). These results suggest constant partial pressure of arterial or end‐tidal CO_2_ during thigh‐cuff deflation trials (Fadel et al., 2001; Ogoh et al., 2008; Ogoh, Tzeng et al., 2010), which was confirmed in one participant who underwent capnometer measurements. Moreover, the duration of period I was equivalent to one breath‐time, suggesting that arterial or end‐tidal CO_2_ was unlikely to change markedly within the time and affect the cerebrovascular responses. Fourth, caution is needed to extrapolate the present findings to females because of probable sex difference in regulating pressure and flow (Barnes, 2017). The same is true of older or clinical populations. Fifth, the relationship between the ability of dynamic cerebral autoregulation and cardiac baroreflex sensitivity was outside the scope of our research. The relationship reported in previous studies was discrepant: inverse correlation (Nasr et al., 2014; Tzeng et al., 2010), no correlation (Aengevaeren et al., 2013), and a positive relationship (i.e., an important role of the cardiac baroreflex in cerebral autoregulation) (Ogoh, Tzeng et al., 2010). This inconsistency may be partly due to differences in participants' characteristics (e.g., age, exercise habits), experimental manipulation, and the way to assess cardiac baroreflex sensitivity. More sophisticated research will be needed to clarify this issue. In addition, assessing whether the efferent limb of carotid sinus baroreflex is involved in the pathway of dynamic cerebral neural autoregulation was also beyond the present research scope. Finally, the role of the aortic baroafferent signals in cerebral autoregulation remains unknown. Taking different anatomical locations and physiological roles between two baroreceptors into account (Ishii et al., 2015; Smith et al., 2001), it may be possible that aortic baroafferent signals play a smaller role in dynamic cerebral autoregulation.

The present study revealed that carotid sinus baroafferent signals accelerate dynamic cerebral autoregulation in the face of rapid hypotension in healthy young males, which has not been reported previously. The neural mechanism appears to constitute approximately 20%–40% of the cerebral autoregulation. Integrating the present findings and the anatomical structure with the old concept for dynamic cerebral autoregulation leads to the developed concept that the neural mechanism and the traditional myogenic mechanism (Claassen et al., 2021) have complementary effects in cerebral autoregulation against a rapid drop in AP. That is, the carotid sinus baroreceptors first detect AP changes to the brain before the intracranial arteries do; the neural mechanism roughly and broadly regulates large cerebral arteries and arterioles (e.g., in the pial circulation) to deliver appropriate blood to the more distal blood vessels. This rough blood flow regulation is further finely adjusted by the myogenic mechanism (e.g., in the brain parenchyma). In such a way, the neural and myogenic mechanisms may synergistically cause dynamic cerebral autoregulation in the face of rapid hypotension.

Impairment of the cerebral neural autoregulation would allow AP‐dependent reduction of cerebral blood flow during standing and would increase the risk of posture‐related syncope (Claydon & Hainsworth, 2003). Frequent occurrence of such cerebral hypoperfusion in daily life potentially augments the synergistic effect of flow reduction and embolization, which could result in stroke (Caplan et al., 2006; Regenhardt et al., 2018). This explanation fits well with carotid stenosis cases: impaired cerebral autoregulation indicates an increased risk of subsequent ischemic events (Howard et al., 2021; Reinhard et al., 2008). Elucidating the mechanism of cerebral neural autoregulation could provide a new target for preventing cerebral ischemic symptoms and diseases.

## AUTHOR CONTRIBUTIONS

K.I. and H.K. conceived and designed the study. K.I., T.I., and R.A. acquired the data. K.I. analyzed the data and drafted the manuscript. All authors interpreted the data, revised the manuscript, provided intellectual feedback, and approved the final manuscript.

## FUNDING INFORMATION

This research was supported in part by Grant‐in‐Aid for Young Scientists (20 K19473) from the Japan Society for the Promotion of Science.

## CONFLICT OF INTEREST STATEMENT

None.

## ETHICS APPROVAL STATEMENT

This study was approved by the Institutional Review Board of the National Institute of Advanced Industrial Science and Technology (HF2016‐0322).

## Supporting information


Data S1.

## References

[phy215937-bib-0001] Aaslid, R. , Lindegaard, K. F. , Sorteberg, W. , & Nornes, H. (1989). Cerebral autoregulation dynamics in humans. Stroke, 20, 45–52. 2492126 10.1161/01.str.20.1.45

[phy215937-bib-0002] Aengevaeren, V. L. , Claassen, J. A. , Levine, B. D. , & Zhang, R. (2013). Cardiac baroreflex function and dynamic cerebral autoregulation in elderly Masters athletes. Journal of Applied Physiology (1985), 114, 195–202. 10.1152/japplphysiol.00402.2012 23139365

[phy215937-bib-0003] Al‐Rawi, P. G. , Smielewski, P. , & Kirkpatrick, P. J. (2001). Evaluation of a near‐infrared spectrometer (NIRO 300) for the detection of intracranial oxygenation changes in the adult head. Stroke, 32, 2492–2500. 11692006 10.1161/hs1101.098356

[phy215937-bib-0004] Barnes, J. N. (2017). Sex‐specific factors regulating pressure and flow. Experimental Physiology, 102, 1385–1392. 28799254 10.1113/EP086531 PMC5665704

[phy215937-bib-0005] Bekar, L. K. , Wei, H. S. , & Nedergaard, M. (2012). The locus coeruleus‐norepinephrine network optimizes coupling of cerebral blood volume with oxygen demand. Journal of Cerebral Blood Flow and Metabolism, 32, 2135–2145. 22872230 10.1038/jcbfm.2012.115 PMC3519408

[phy215937-bib-0006] Brassard, P. , Roy, M. A. , Burma, J. S. , Labrecque, L. , & Smirl, J. D. (2023). Quantification of dynamic cerebral autoregulation: Welcome to the jungle. Clinical Autonomic Research, 33, 791–810. 37758907 10.1007/s10286-023-00986-2

[phy215937-bib-0007] Briggs, L. , Garcia, J. H. , Conger, K. A. , Pinto de Moraes, H. , Geer, J. C. , & Hollander, W. (1985). Innervation of brain intraparenchymal vessels in subhuman primates: Ultrastructural observations. Stroke, 16, 297–301. 3975968 10.1161/01.str.16.2.297

[phy215937-bib-0008] Caplan, L. R. , Wong, K. S. , Gao, S. , & Hennerici, M. G. (2006). Is hypoperfusion an important cause of strokes? If so, how. Cerebrovascular Diseases, 21, 145–153. 16401883 10.1159/000090791

[phy215937-bib-0009] Claassen, J. , Thijssen, D. H. J. , Panerai, R. B. , & Faraci, F. M. (2021). Regulation of cerebral blood flow in humans: Physiology and clinical implications of autoregulation. Physiological Reviews, 101, 1487–1559. 33769101 10.1152/physrev.00022.2020 PMC8576366

[phy215937-bib-0010] Claydon, V. E. , & Hainsworth, R. (2003). Cerebral autoregulation during orthostatic stress in healthy controls and in patients with posturally related syncope. Clinical Autonomic Research, 13, 321–329. 14564654 10.1007/s10286-003-0120-8

[phy215937-bib-0011] Cohen, Z. , Molinatti, G. , & Hamel, E. (1997). Astroglial and vascular interactions of noradrenaline terminals in the rat cerebral cortex. Journal of Cerebral Blood Flow and Metabolism, 17, 894–904. 9290587 10.1097/00004647-199708000-00008

[phy215937-bib-0012] Coverdale, N. S. , Lalande, S. , Perrotta, A. , & Shoemaker, J. K. (2015). Heterogeneous patterns of vasoreactivity in the middle cerebral and internal carotid arteries. American Journal of Physiology. Heart and Circulatory Physiology, 308, H1030–H1038. 25724496 10.1152/ajpheart.00761.2014

[phy215937-bib-0013] Dwan, K. , Li, T. , Altman, D. G. , & Elbourne, D. (2019). CONSORT 2010 statement: Extension to randomised crossover trials. BMJ, 366, l4378. 31366597 10.1136/bmj.l4378 PMC6667942

[phy215937-bib-0014] Eckberg, D. L. (1976). Temporal response patterns of the human sinus node to brief carotid baroreceptor stimuli. The Journal of Physiology, 258, 769–782. 978502 10.1113/jphysiol.1976.sp011445 PMC1309004

[phy215937-bib-0015] Edvinsson, L. , Owman, C. , & Sjoberg, N. O. (1976). Autonomic nerves, mast cells, and amine receptors in human brain vessels. A histochemical and pharmacological study. Brain Research, 115, 377–393. 184880 10.1016/0006-8993(76)90356-5

[phy215937-bib-0016] Fadel, P. J. , Stromstad, M. , Hansen, J. , Sander, M. , Horn, K. , Ogoh, S. , Smith, M. L. , Secher, N. H. , & Raven, P. B. (2001). Arterial baroreflex control of sympathetic nerve activity during acute hypotension: Effect of fitness. American Journal of Physiology. Heart and Circulatory Physiology, 280, H2524–H2532. 11356607 10.1152/ajpheart.2001.280.6.H2524

[phy215937-bib-0017] Giller, C. A. , Bowman, G. , Dyer, H. , Mootz, L. , & Krippner, W. (1993). Cerebral arterial diameters during changes in blood pressure and carbon dioxide during craniotomy. Neurosurgery, 32, 737–741. 8492848

[phy215937-bib-0018] Hamner, J. W. , Tan, C. O. , Lee, K. , Cohen, M. A. , & Taylor, J. A. (2010). Sympathetic control of the cerebral vasculature in humans. Stroke, 41, 102–109. 20007920 10.1161/STROKEAHA.109.557132 PMC2814242

[phy215937-bib-0019] Hamner, J. W. , Tan, C. O. , Tzeng, Y. C. , & Taylor, J. A. (2012). Cholinergic control of the cerebral vasculature in humans. The Journal of Physiology, 590, 6343–6352. 23070700 10.1113/jphysiol.2012.245100 PMC3533196

[phy215937-bib-0020] Heistad, D. D. , & Marcus, M. L. (1976). Total and regional cerebral blood flow during stimulation of carotid baroreceptors. Stroke, 7, 239–243. 942613 10.1161/01.str.7.3.239

[phy215937-bib-0021] Heistad, D. D. , & Marcus, M. L. (1978). Evidence that neural mechanisms do not have important effects on cerebral blood flow. Circulation Research, 42, 295–302. 203412 10.1161/01.res.42.3.295

[phy215937-bib-0022] Hosford, P. S. , & Gourine, A. V. (2019). What is the key mediator of the neurovascular coupling response? Neuroscience and Biobehavioral Reviews, 96, 174–181. 30481531 10.1016/j.neubiorev.2018.11.011 PMC6331662

[phy215937-bib-0023] Hotta, H. , Masamoto, K. , Uchida, S. , Sekiguchi, Y. , Takuwa, H. , Kawaguchi, H. , Shigemoto, K. , Sudo, R. , Tanishita, K. , Ito, H. , & Kanno, I. (2013). Layer‐specific dilation of penetrating arteries induced by stimulation of the nucleus basalis of Meynert in the mouse frontal cortex. Journal of Cerebral Blood Flow and Metabolism, 33, 1440–1447. 23756692 10.1038/jcbfm.2013.92 PMC3764390

[phy215937-bib-0024] Howard, D. P. J. , Gaziano, L. , Rothwell, P. M. , & Oxford, V. S. (2021). Risk of stroke in relation to degree of asymptomatic carotid stenosis: A population‐based cohort study, systematic review, and meta‐analysis. Lancet Neurology, 20, 193–202. 33609477 10.1016/S1474-4422(20)30484-1 PMC7889579

[phy215937-bib-0025] Hu, H. H. , Kuo, T. B. , Wong, W. J. , Luk, Y. O. , Chern, C. M. , Hsu, L. C. , & Sheng, W. Y. (1999). Transfer function analysis of cerebral hemodynamics in patients with carotid stenosis. Journal of Cerebral Blood Flow and Metabolism, 19, 460–465. 10197516 10.1097/00004647-199904000-00012

[phy215937-bib-0026] Ishii, K. , Idesako, M. , & Matsukawa, K. (2015). Differential contribution of aortic and carotid sinus baroreflexes to control of heart rate and renal sympathetic nerve activity. The Journal of Physiological Sciences, 65, 471–480. 26159318 10.1007/s12576-015-0387-2 PMC10717140

[phy215937-bib-0027] Ishii, K. , Liang, N. , Asahara, R. , Takahashi, M. , & Matsukawa, K. (2018). Feedforward‐ and motor effort‐dependent increase in prefrontal oxygenation during voluntary one‐armed cranking. The Journal of Physiology, 596, 5099–5118. 30175404 10.1113/JP276956 PMC6209741

[phy215937-bib-0028] Kawai, Y. (2018). Differential ascending projections from the male rat caudal nucleus of the Tractus solitarius: An interface between local microcircuits and global macrocircuits. Frontiers in Neuroanatomy, 12, 63. 30087599 10.3389/fnana.2018.00063 PMC6066510

[phy215937-bib-0029] Kober, G. , & Arndt, J. O. (1970). Pressure‐diameter relationship in the common carotid artery of conscious man. Pflügers Archiv, 314, 27–39. 5460688 10.1007/BF00587044

[phy215937-bib-0030] Labrecque, L. , Rahimaly, K. , Imhoff, S. , Paquette, M. , Le Blanc, O. , Malenfant, S. , Lucas, S. J. E. , Bailey, D. M. , Smirl, J. D. , & Brassard, P. (2017). Diminished dynamic cerebral autoregulatory capacity with forced oscillations in mean arterial pressure with elevated cardiorespiratory fitness. Physiological Reports, 5, 13486. 10.14814/phy2.13486 PMC5688778 29122957

[phy215937-bib-0031] Labrecque, L. , Smirl, J. D. , Tzeng, Y. C. , & Brassard, P. (2022). Point/counterpoint: We should take the direction of blood pressure change into consideration for dynamic cerebral autoregulation quantification. Journal of Cerebral Blood Flow and Metabolism, 42, 2351–2353. 35619230 10.1177/0271678X221104868 PMC9670010

[phy215937-bib-0032] Lind‐Holst, M. , Cotter, J. D. , Helge, J. W. , Boushel, R. , Augustesen, H. , Van Lieshout, J. J. , & Pott, F. C. (2011). Cerebral autoregulation dynamics in endurance‐trained individuals. Journal of Applied Physiology (1985), 110, 1327–1333. 10.1152/japplphysiol.01497.2010 21372098

[phy215937-bib-0033] Murkin, J. M. , Baird, D. L. , Martzke, J. S. , & Yee, R. (1997). Cognitive dysfunction after ventricular fibrillation during implantable cardiovertor/defibrillator procedures is related to duration of the reperfusion interval. Anesthesia and Analgesia, 84, 1186–1192. 9174290 10.1097/00000539-199706000-00003

[phy215937-bib-0034] Nakai, M. , & Ogino, K. (1984). The relevance of cardio‐pulmonary‐vascular reflex to regulation of the brain vessels. The Japanese Journal of Physiology, 34, 193–197. 6727069 10.2170/jjphysiol.34.193

[phy215937-bib-0035] Nasr, N. , Czosnyka, M. , Pavy‐Le Traon, A. , Custaud, M. A. , Liu, X. , Varsos, G. V. , & Larrue, V. (2014). Baroreflex and cerebral autoregulation are inversely correlated. Circulation Journal, 78, 2460–2467. 25187067 10.1253/circj.cj-14-0445

[phy215937-bib-0036] Ogoh, S. , Brothers, R. M. , Eubank, W. L. , & Raven, P. B. (2008). Autonomic neural control of the cerebral vasculature: Acute hypotension. Stroke, 39, 1979–1987. 18451346 10.1161/STROKEAHA.107.510008

[phy215937-bib-0037] Ogoh, S. , Sato, K. , Akimoto, T. , Oue, A. , Hirasawa, A. , & Sadamoto, T. (2010). Dynamic cerebral autoregulation during and after handgrip exercise in humans. Journal of Applied Physiology (1985), 108, 1701–1705. 10.1152/japplphysiol.01031.2009 20378708

[phy215937-bib-0038] Ogoh, S. , & Tarumi, T. (2019). Cerebral blood flow regulation and cognitive function: A role of arterial baroreflex function. The Journal of Physiological Sciences, 69, 813–823. 31444691 10.1007/s12576-019-00704-6 PMC10717347

[phy215937-bib-0039] Ogoh, S. , Tzeng, Y. C. , Lucas, S. J. , Galvin, S. D. , & Ainslie, P. N. (2010). Influence of baroreflex‐mediated tachycardia on the regulation of dynamic cerebral perfusion during acute hypotension in humans. The Journal of Physiology, 588, 365–371. 19933752 10.1113/jphysiol.2009.180844 PMC2821730

[phy215937-bib-0040] Panerai, R. B. , Barnes, S. C. , Batterham, A. P. , Robinson, T. G. , & Haunton, V. J. (2023). Directional sensitivity of dynamic cerebral autoregulation during spontaneous fluctuations in arterial blood pressure at rest. Journal of Cerebral Blood Flow and Metabolism, 43, 552–564. 36420777 10.1177/0271678X221142527 PMC10063834

[phy215937-bib-0041] Regenhardt, R. W. , Das, A. S. , Lo, E. H. , & Caplan, L. R. (2018). Advances in understanding the pathophysiology of lacunar stroke: A review. JAMA Neurology, 75, 1273–1281. 30167649 10.1001/jamaneurol.2018.1073 PMC7426021

[phy215937-bib-0042] Reinhard, M. , Gerds, T. A. , Grabiak, D. , Zimmermann, P. R. , Roth, M. , Guschlbauer, B. , Timmer, J. , Czosnyka, M. , Weiller, C. , & Hetzel, A. (2008). Cerebral dysautoregulation and the risk of ischemic events in occlusive carotid artery disease. Journal of Neurology, 255, 1182–1189. 18575926 10.1007/s00415-008-0865-z

[phy215937-bib-0043] Reinhard, M. , Roth, M. , Muller, T. , Guschlbauer, B. , Timmer, J. , Czosnyka, M. , & Hetzel, A. (2004). Effect of carotid endarterectomy or stenting on impairment of dynamic cerebral autoregulation. Stroke, 35, 1381–1387. 15087557 10.1161/01.STR.0000127533.46914.31

[phy215937-bib-0044] Sagawa, K. , & Guyton, A. C. (1961). Pressure‐flow relationships in isolated canine cerebral circulation. The American Journal of Physiology, 200, 711–714. 13745365 10.1152/ajplegacy.1961.200.4.711

[phy215937-bib-0045] Sanders, J. S. , Mark, A. L. , & Ferguson, D. W. (1989). Importance of aortic baroreflex in regulation of sympathetic responses during hypotension. Evidence from direct sympathetic nerve recordings in humans. Circulation, 79, 83–92. 2910547 10.1161/01.cir.79.1.83

[phy215937-bib-0046] Smith, S. A. , Querry, R. G. , Fadel, P. J. , Weiss, M. W. , Olivencia‐Yurvati, A. , Shi, X. , & Raven, P. B. (2001). Comparison of aortic and carotid baroreflex stimulus‐response characteristics in humans. Autonomic Neuroscience, 88, 74–85. 11474549 10.1016/S1566-0702(01)00214-4

[phy215937-bib-0047] Suzuki, N. , Hardebo, J. E. , & Owman, C. (1989). Origins and pathways of cerebrovascular nerves storing substance P and calcitonin gene‐related peptide in rat. Neuroscience, 31, 427–438. 2477770 10.1016/0306-4522(89)90385-0

[phy215937-bib-0048] Tzeng, Y. C. , Lucas, S. J. , Atkinson, G. , Willie, C. K. , & Ainslie, P. N. (2010). Fundamental relationships between arterial baroreflex sensitivity and dynamic cerebral autoregulation in humans. Journal of Applied Physiology (1985), 108, 1162–1168. 10.1152/japplphysiol.01390.2009 20223996

[phy215937-bib-0049] White, R. P. , & Markus, H. S. (1997). Impaired dynamic cerebral autoregulation in carotid artery stenosis. Stroke, 28, 1340–1344. 9227680 10.1161/01.str.28.7.1340

[phy215937-bib-0050] Xiong, L. , Liu, X. , Shang, T. , Smielewski, P. , Donnelly, J. , Guo, Z. N. , Yang, Y. , Leung, T. , Czosnyka, M. , Zhang, R. , Liu, J. , & Wong, K. S. (2017). Impaired cerebral autoregulation: Measurement and application to stroke. Journal of Neurology, Neurosurgery, and Psychiatry, 88, 520–531. 28536207 10.1136/jnnp-2016-314385

[phy215937-bib-0051] Zhang, R. , Zuckerman, J. H. , Iwasaki, K. , Wilson, T. E. , Crandall, C. G. , & Levine, B. D. (2002). Autonomic neural control of dynamic cerebral autoregulation in humans. Circulation, 106, 1814–1820. 12356635 10.1161/01.cir.0000031798.07790.fe

[phy215937-bib-0052] Zunker, P. , Haase, C. , Borggrefe, M. , Georgiadis, D. , Georgiadis, A. , & Ringelstein, E. B. (1998). Cerebral hemodynamics during induced tachycardia in routine electrophysiologic studies: A transcranial doppler study. Neurological Research, 20, 504–508. 9713840 10.1080/01616412.1998.11740555

