# Supplementary material for: Carotid sinus baroafferent signals contribute to cerebral blood flow regulation during acute hypotension in young males: A randomized crossover study
Source: Physiol Rep. 2024 Feb 7;12(3):e15937. doi: 10.14814/phy2.15937 (PMC10849886; doi:10.14814/phy2.15937)
Supplement: Supplementary file 1 — Data S1. [file PHY2-12-e15937-s001.pdf]

Supplementary Figure

Effects of ipsilateral arm suction on brachial and radial artery blood flows

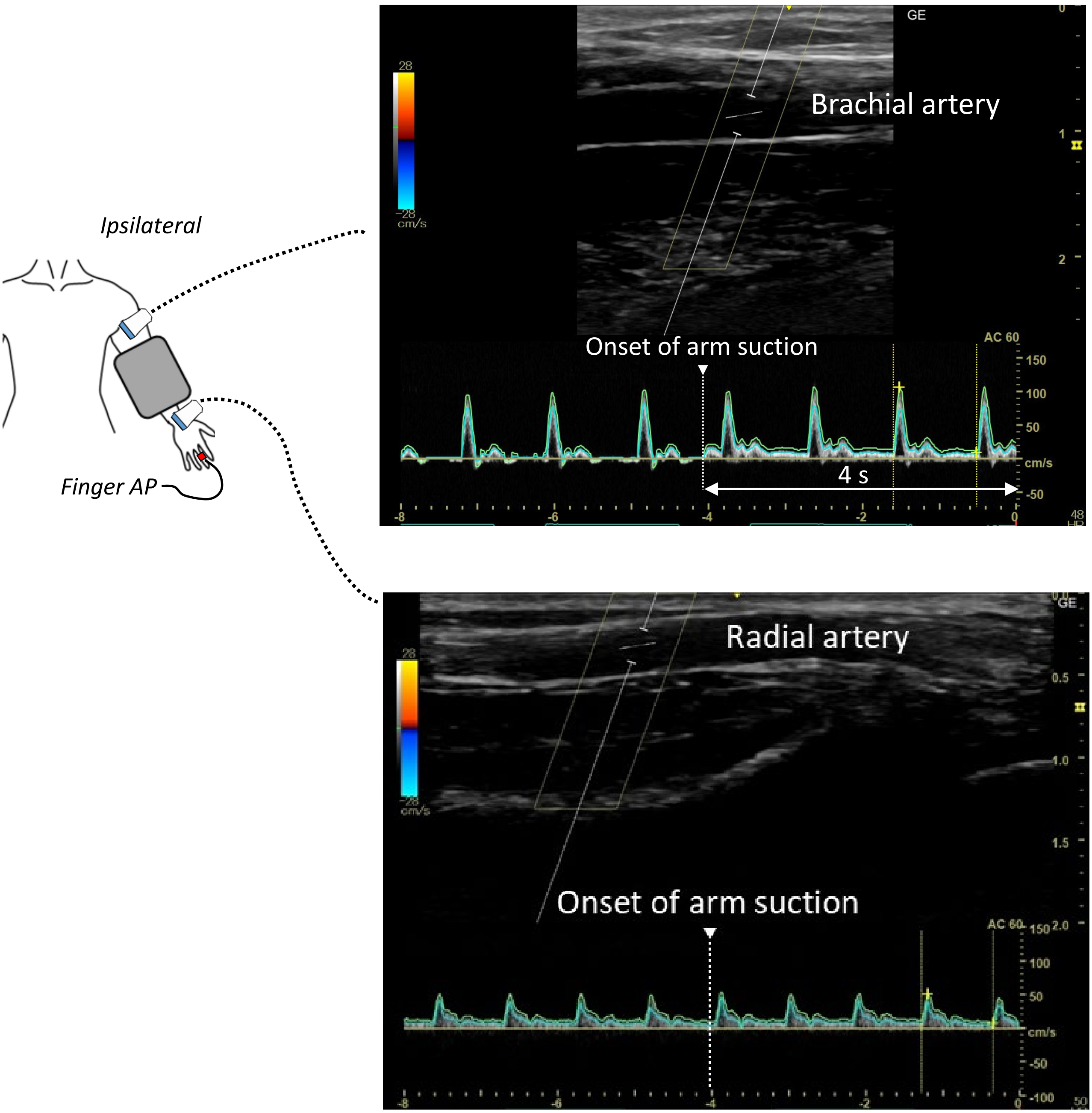

Ipsilateral arm suction increased brachial artery blood flow velocity as reported previously (Holder et al., *J Appl Physiol* 126: 1687–1693, 2019), while it did not affect radial artery blood flow velocity. The diameter of each artery did not change from the pre-suction level (*data not shown*).
